# Values of serum neutrophil gelatinase-associated lipocalin and cystatin C after percutaneous coronary intervention for early diagnosis of contrast-induced nephropathy

**DOI:** 10.4314/ahs.v23i3.69

**Published:** 2023-09

**Authors:** Ping Luo, Wei Ao, Dikai Xiang, Jian Wang, Jia Liu

**Affiliations:** Department of Cardiology, Yueyang People's Hospital, Yueyang 414000, Hunan Province, China

**Keywords:** Contrast, cystatin C, nephropathy, neutrophil gelatinase-associated lipocalin, percutaneous coronary intervention

## Abstract

**Objective:**

Serum creatinine (SCr) is not a sensitive and reliable index for the early diagnosis of acute kidney injury caused by contrast-induced nephropathy (CIN). The aim of this study was to explore the values of serum neutrophil gelatinase-associated lipocalin (NGAL) and cystatin C (Cys-C) after percutaneous coronary intervention (PCI) for the early diagnosis of CIN.

**Methods:**

Three hundred patients receiving PCI from January 2018 to December 2020 were assigned to a CIN group (n=25) and a non-CIN group (n=275), respectively. SCr, Cys-C and NGAL levels were measured, and their sensitivities for early CIN diagnosis were evaluated by the area under the receiver operating characteristic curve (AUC) values.

**Results:**

The NGAL and Cys-C levels of the CIN group began to rise 6 and 12 h after operation, respectively (P<0.05). The CIN group had higher NGAL and Cys-C levels than those of the non-CIN group 12, 24 and 48 h after operation (P<0.05). The AUC values of NGAL, Cys-C and SCr 24 h after operation were 0.885, 0.874 and 0.856, respectively.

**Conclusion:**

The serum NGAL and Cys-C levels of patients after PCI reflect the early changes of renal function, which are valuable for early CIN diagnosis.

## Introduction

Interventional technology has been extensively applied to the diagnosis and treatment of diseases. However, it frequently causes contrast-induced nephropathy (CIN). The number of CIN-induced acute kidney injury (AKI) cases accounts for 11 percent of the total number of AKI cases. CIN refers to the iatrogenic renal function damage induced by contrast media, but it has elusive pathogenesis and no specific diagnosis or treatment method^1^-^3^. Early clinical diagnosis is the key to CIN prevention, which can significantly improve the prognosis of patients in combination with appropriate therapies. Currently, CIN is commonly diagnosed by detecting the levels of serum creatinine (SCr) and blood urea nitrogen. Nevertheless, SCr level is influenced by a variety of internal and external factors such as age, gender, diet, weight and nutritional status^4^. Hence, SCr is not a sensitive and reliable index for the early diagnosis of AKI.

As a member of the lipocalin family, NGAL is widely distributed in various tissues, such as the bronchus, gastrointestinal tract and renal tubules. NGAL can induce renal progenitor cells to differentiate into renal tubular epithelial cells. Once renal tubules are damaged, the serum level of NGAL rises for repair and regeneration^5^. It has been proven that serum neutrophil gelatinase-associated lipocalin (NGAL) and cystatin C (Cys-C) can predict AKI earlier^6^.

Thereby motivated, the serum NGAL and Cys-C levels of patients undergoing PCI were detected at different time points before and after operation in this study, aiming to explore the values of these indices for the early diagnosis of CIN.

## Materials and methods

### Subjects and baseline clinical data

This study has been approved by the ethics committee of Yueyang Second People's Hospital, and written informed consents have been obtained from all patients. A total of 300 patients receiving PCI in Yueyang Second People's Hospital from January 2018 to December 2020 were enrolled as subjects of this prospective study. The subjects consisted of 166 males and 134 females who were aged (61.03±5.12) years old.

All the patients met the diagnostic criteria for coronary heart disease and indications for PCI. The exclusion criteria were as follows: (1) Patients with AKI caused by pre-renal, renal or postrenal obstruction, (2) those who used nephrotoxic drugs or contrast media within 2 weeks before operation, (3) those with severe heart failure or other organ failure, (4) those with acute myocardial infarction, or (5) those with acute infection. CIN was defined as elevation in the SCr level by 0.5 mg/dL (44 µmol/L) or 25 percent in comparison with the baseline value within 72 h after intravascular injection of iodinated contrast media, with other causes excluded.

### Main reagents and apparatus

NGAL level was detected by enzyme-linked immunosorbent assay (ELISA) kit and related reagents (Beijing Hotgen Biotech Co., Ltd., China) using HDF-7080 automatic biochemical analyzer (Hitachi, Japan). Cys-C level was determined by rate nephelometry and SCr level was measured with the picric acid rate method using reagents provided by Anhui Daqian Bio-engineering Co., Ltd. (China).

### Sample Collection

A total of 5 mL of venous blood was collected from each subject, and added in an anticoagulant tube containing EDTA. After centrifugation at 4,000 rpm for 10 min, 1.5 mL of serum was added in an aseptic EP tube which was then marked and stored in a refrigerator at -80°C.

### Sample detection

Serum NGAL level was detected by ELISA. In detail, samples and control were added into the wells of plates that were sealed for incubation at 37°C for 120 min. After the liquid was discarded, reaction solution A was added into each well for incubation at 37°C for 60 min. Then the plate was washed and pat-dried (repeated 5 times), and added reaction solution B for incubation at 37°C for 60 min. Subsequently, the plate was washed again, and colour development solution was added into each well for incubation at 37°C for 10 min. After stop buffer was added into each well, the optical density at 450 nm was measured. Finally, the corresponding concentration of sample was calculated based on the plotted standard curve.

### Statistical analysis

SPSS 26.0 software (IBM Inc., USA) was utilized for statistical analysis. MedCalc software (version 11.5.1.0; Med-Calc Software, Belgium) was employed to compare the receiver operating characteristic (ROC) curves at different time points. The measurement data in line with normal distribution were expressed as mean ± standard deviation, and examined by the t-test for multigroup comparisons. The χ^2^ test was performed for comparison between rates. The Mann-Whitney rank sum test was employed for non-normally distributed data. P<0.05 suggested that a difference was statistically significant.

## Results

### Clinical data of CIN and non-CIN groups

Among the 300 patients, 25 (8.33%) suffered from CIN, including 17 males and 8 females who were aged (62.31±5.21) years old. Among the 25 CIN patients, there were 8 cases of hypertension, 12 cases of diabetes mellitus and 5 cases of hyperlipidemia. Compared with the non-CIN group, the CIN group had more cases of diabetes mellitus and longer hospitalization time (P<0.05). However, the two groups had similar numbers of hypertension and hyperlipidemia cases, contrast medium dose, and SCr, NGAL and Cys-C levels upon admission (P>0.05) (Table 1). Collectively, CIN patients were more prone to complication with diabetes mellitus, accompanied by severer symptoms and longer treatment time than non-CIN cases.

**Table 1:** Clinical data of CIN and non-CIN groups

Index	CIN group (n=25)	Non-CIN group (n=275)	Statistical value	P
Gender			χ^2^=1.396	0.237
Male	17 (68.00)	159		
Female	8	126		
Age (Y)	62.31±5.21	61.03±5.12	t=1.195	0.233
Diabetes mellitus [n (%)]	13	54	χ^2^=16.795	0.000
Hypertension [n (%)]	8	86	χ^2^=0.006	0.940
Hyperlipidemia [n (%)]	5	54	χ^2^=0.002	0.965
Contrast medium dose (mL)	201.86±26.36	199.56±25.43	t=0.432	0.666
Hospitalization time (d)	16.31±2.34	9.82±0.74	t=31.969	0.000
SCr upon admission (µmol/L)	68.27±5.78	66.54±6.02	t=1.380	0.169
NGAL upon admission (ng/mL)	87.43±11.34	86.95±10.28	t=0.222	0.825
Cys-C upon admission (mg/L)	0.98±0.11	0.96±0.09	t=1.043	0.149

### SCr, NGAL and Cys-C levels of CIN and non-CIN groups at different time points after PCI

The serum NGAL level of the CIN group began to rise 6 h after operation, while the serum Cys-C level started to increase 12 h after operation and peaked 12-24 h after operation. Both levels began to decline 48 h after operation. The serum levels of NGAL and Cys-C in the CIN group were significantly higher than those of the non-CIN group 12, 24 and 48 h after operation (P<0.05). However, the two groups had similar levels of SCr and Cys-C 6 h after operation (P>0.05) (Table 2). Taken together, the SCr, NGAL, and Cys-C levels of patients after PCI increased first and then decreased, and NGAL level changed earlier. Additionally, the NGAL and CysC levels of CIN patients were higher than those of non-CIN patients 12, 24 and 48 h after surgery.

**Table 2:** SCr, NGAL and Cys-C levels of CIN and non-CIN groups at different time points after PCI

Group	SCr (µmol/L)				
	0 h	6 h	12 h	24 h	48 h

CIN group (n=25)	65.38±5.76	71.88±8.23	85.79±8.23	108.29±13.24	119.27±14.27
Non-CIN group (n=275)	67.79±6.34	69.27±9.25	71.28±9.65	70.95±8.27	73.28±9.28
Intergroup	F=119.276, P=0.000				
Time point	F=67.276, P=0.000				
Intergroup × Time point	F=27.387, P=0.000				
	NGAL (ng/mL)				

	0 h	6 h	12 h	24 h	48 h

CIN group (n=25)	86.73±9.28	126.34±11.34	171.28±24.38	165.98±23.24	123.27±24.21
Non-CIN group (n=275)	88.25±9.23	88.04±8.46	88.56±9.04	89.25±8.27	87.45±7.82
Intergroup	F=432.872, P=0.000				
Time point	F=56.294, P=0.000				
Intergroup × Time point	F=45.281, P=0.000				
	CysC (mg/L)				

	0 h	6 h	12 h	24 h	48h

CIN group (n=25)	0.94±0.11	1.27±0.11	1.96±0.25	2.02±0.26	1.24±0.11
Non-CIN group (n=275)	0.96±0.09	1.05±0.12	1.02±0.23	1.03±0.11	1.04±0.09
Intergroup	F=134.298, P=0.000				
Time point	F=34.264, P=0.000				
Intergroup × Time point	F=25.43, P=0.000				

### AUC values of serum NGAL and Cys-C and SCr at different time points after PCI

To determine the sensitivity and specificity of serum NGAL and Cys-C and SCr for the diagnosis of CIN at different time points after PCI, the areas under the ROC curves (AUCs) were calculated with the detection results as the cut-off values. AUCs of NGAL, Cys-C and SCr 6 h after operation were 0.824, 0.753 and 0.584, respectively, those 12 h after operation were 0.902, 0.842 and 0.802, respectively, those 24 h after operation were 0.885, 0.874 and 0.856, respectively, and those 48 h after operation were 0.774, 0.702 and 0.884, respectively. The AUC values of serum NGAL and Cys-C were higher than those of SCr 6, 12 and 24 h after PCI, but the values of NGAL and Cys-C were lower than that of SCr 48 h after PCI. In a word, the specificity and sensitivity of NGAL reached maxima 12 and 24 h after PCI (Table 3).

**Table 3:** AUCs of serum NGAL and Cys-C and SCr at different time points after PCI

Time	Item	AUC	Sensitivity	Specificity	Cut-off	P	95% CI
6 h after operation	SCr (µmol/L)	0.584	62.54	58.29	73.02	0.382	0.462~0.663
	NGAL (ng/mL)	0.824	98.27	67.02	96.24	0.002	0.729~0.879
	CysC (mg/L)	0.753	61.78	92.38	1.12	0.009	0.664~0.824
12 h after operation	SCr (µmol/L)	0.802	68.22	83.42	78.04	<0.001	0.712~0.883
	NGAL (ng/mL)	0.902	95.27	75.06	94.62	<0.001	0.816~0.943
	CysC (mg/L)	0.842	66.82	98.43	1.13	<0.001	0.753~0.893
24 h after operation	SCr (µmol/L)	0.856	59.49	97.82	73.22	<0.001	0.784~0.902
	NGAL (ng/mL)	0.885	98.26	67.34	98.03	<0.001	0.803~0.929
	CysC (mg/L)	0.874	62.34	91.72	1.08	<0.001	0.785~0.911
48 h after operation	SCr (µmol/L)	0.884	89.26	75.09	88.21	<0.001	0.802~0.923
	NGAL (ng/mL)	0.774	93.46	66.87	96.04	<0.001	0.683~0.852
	CysC (mg/L)	0.702	59.29	99.28	1.18	<0.001	0.604~0.784

There was a significant difference between the ROC curves of SCr and NGAL 6 h after operation (P=0.039), but no significant differences were found between the curves of SCr and Cys-C (P=0.071) or between those of Cys-C and NGAL (P=0.656). There were no significant differences between the ROC curves of SCr and NGAL (P=0.286), between those of SCr and Cys-C (P=0.672) or between those of Cys-C and NGAL (P=0.405) 12 h after operation. No significant differences were found between the ROC curves of SCr and NGAL (P=0.743), between those of SCr and Cys-C (P=0.983) or between those of Cys-C and NGAL (P=0.754) 24 h after operation. Nevertheless, there was a significant difference between the ROC curves of SCr and Cys-C (P=0.024) 48 h after operation, but no significant differences were observed between the ROC curves of SCr and NGAL (P=0.172) or between those of Cys-C and NGAL (P=0.503). In short, detecting the NGAL levels 12 and 24 h after surgery had higher early diagnostic value for CIN.

## Discussion

The serum NGAL level has been utilized as a marker for the early diagnosis of AKI7. CIN has become one of the common causes for nosocomial AKI. Nevertheless, whether NGAL can be used in the early diagnosis of CIN remains largely unknown. Under normal conditions, NGAL is lowly expressed in the kidneys, stomach and intestines of human body. When AKI occurs, renal tubular epithelial cells are damaged, so NGAL is released in large quantities and can be quickly detected in the blood and urine. In the case of CIN, NGAL level rises after 2 h, which can help the early diagnosis of renal function damage after coronary angiography and interventional therapy.

Ariza *et al.* reported that NGAL level increased in the blood and tissues of CIN mice 2 h after the onset of CIN, while SCr level rose 48 h after onset^8^. In CIN children, the serum NGAL level increases 2 h after the use of contrast medium, while SCr level is elevated 12-24 h after administration. The NGAL level of CIN patients is raised 8 h after operation, while SCr level is augmented 24 h after operation. The ROC curves show that the sensitivity and specificity of serum and urine NGAL levels are as high as 0.995 and 0.992, respectively^9^. It is well-documented that serum or urine NGAL level has high specificity and sensitivity for the early diagnosis of CIN^10^,^11^. Moreover, the NGAL level of the CIN group rises 6 h after PCI^12^. Filiopoulos et al. found that the serum NGAL level began to rise in CIN patients 6 h after CT angiography. The above studies suggest that serum NGAL is more sensitive than SCr for the early diagnosis of CIN^13^. In the present study, the serum NGAL level of the CIN group began to rise 6 h after operation, while the serum Cys-C level started to increase 12 h after operation and peaked 12-24 h after operation. Both levels began to decline 48 h after operation. Moreover, SCr level hardly increased 6 h after operation.

As an alkaline non-glycosylated protein with the relative molecular weight of 13,300, Cys-C can penetrate the glomerular filtration membrane freely. Cys-C is not secreted by renal tubules, but reabsorbed and catabolized by proximal tubules. The product does not return to the blood. Hence, the injury of glomeruli and renal tubules can be assessed by detecting Cys-C level. Serum Cys-C has high sensitivity and specificity for both acute and chronic renal damage^14^-^17^. Currently, Cys-C has been applied in clinical practice for the early diagnosis of acute and chronic renal failure. However, diagnosing early CIN on the basis of Cys-C level is still controversial. The increase of Cys-C level over 5 percent of the baseline value has a high predictive value for the long-term poor prognosis of CIN patients^18^. The serum NGAL level rises 6 h after the use of contrast medium for CIN children, while Cys-C level increases 24 h after administration^19^. Xie *et al.* reported that CIN was diagnosed 24 h earlier by serum NGAL and Cys-C levels than by SCr level^20^. However, Cecchi *et al.* found that the serum and urine NGAL levels in elderly patients after PCI barely increased, whereas Cys-C level significantly rose^21^. The results of this study demonstrated that AUCs of serum NGAL and Cys-C levels were highest 6, 12 and 24 h after PCI, so Cys-C was an ideal index for the early diagnosis of CIN. However, the serum Cys-C level declined rapidly 48 h after operation.

## Conclusion

In conclusion, the combined detection of NGAL and Cys-C can improve the specificity and sensitivity for the diagnosis of CIN. However, this study still has limitations. Given that the occurrence of CIN is influenced by primary disease, age and contrast medium dose, the sample size of this single-center study is not large enough. In the future, it is necessary to enlarge the sample size to further evaluate the effects of serum NGAL and Cys-C levels on the early diagnosis of CIN.
